# Developing, Modifying, and Validating a TaqMan Real-Time PCR Technique for Accurate Identification of *Leishmania* Parasites Causing Most Leishmaniasis in Iran

**DOI:** 10.3389/fcimb.2021.731595

**Published:** 2021-10-12

**Authors:** Reza Fotouhi-Ardakani, Seyedeh Maryam Ghafari, Paul Donald Ready, Parviz Parvizi

**Affiliations:** ^1^ Cellular and Molecular Research Center, Qom University of Medical Sciences, Qom, Iran; ^2^ Molecular Systematics Laboratory, Parasitology Department, Pasteur Institute of Iran, Tehran, Iran; ^3^ Disease Control Department, London School of Hygiene and Tropical Medicine, London, United Kingdom

**Keywords:** *Leishmania* parasites, AAP3, COII, TaqMan-qPCR, validation

## Abstract

Many laboratory methods are used to diagnose leishmaniasis because it is characterized by varied symptoms and caused by different *Leishmania* species. A quantitative real-time PCR method based on a TaqMan probe was developed and modified for accurate identification of human cutaneous leishmaniasis (caused by *Leishmania major* or *Leishmania tropica*) from endemic areas of Iran. Two gene regions of amino acid permease 3 (AAP3) and cytochrome oxidase II (COII) were considered. Six new sets of species-specific primers and probes were designed. A total of 123 samples were examined and employed to evaluate and validate real-time PCR. According to parasitic load of the genesig^®^ *Leishmania* Advanced Standard Kit, a serial dilution of purified plasmid (2–2×10^7^ copies/reaction) was prepared under the same conditions for both genes. Specific primers and probes were able to detect three and six parasite copies in AAP3 and COII genes, respectively, and were able to detect three copies of parasites for *L. major* and *L. tropica*. The sensitivities of the reference kit and our method were 98.7 and 98.1%, respectively, and specificity was 100% for detecting parasite genomes in all assays. Designed primers and probes performed well in terms of efficiency and regression coefficient. For AAP3 and COII genes, respectively, the linear log range was 7 and the correlation coefficient (R^2^) was 0.749 and 0.996 for the reference kit using the standard generated curve and 0.98 and 0.96 with serial dilutions of parasite DNA. This research detected *L. major* and *L. tropica* definitely and opens the horizon for the other scientists in the multiplex reactions in designing and optimization of the conditions *in silico* and *in vivo*.

## Introduction

The genus *Leishmania* belongs to the flagellate protozoan parasites which are transmitted through the bite of infected female phlebotomine sand flies (Ready, 2013; World Health Organization, 2021) Different manifestations of leishmaniasis depend on *Leishmania* species and genetics and immune status of host and can cause a variety of chronic cutaneous, cutaneous-mucosal, and visceral infections (World Health Organization, 2021). So far, about 20 species of *Leishmania* have been identified worldwide (Dowlati, 1996; World Health Organization, 2021).

Cutaneous leishmaniasis (CL) is usually caused by *L. major*, *L. tropica*, and *L. aetiopica* in the Eastern Hemisphere (Dowlati, 1996; Schönian et al., 2003; Desjeux, 2004; World Health Organization, 2021). It is estimated that about 90% of CL occurs in about seven countries including Iran (Dowlati, 1996; Desjeux, 2004; World Health Organization, 2021). Each year approximately 20,000 CL cases are reported from endemic regions of Iran. CL in Iran is observed in two zoonotic forms: dry (urban) and wet (rural). Wet lesions (rural CL) caused by *L. major* mainly appear on the lower limbs of the body, which include more than 70% of cases of CL in Iran. Dry lesions (urban CL) caused by *L. tropica* usually occur around a healed lesion in new papules (Dowlati, 1996; Tashakori et al., 2003; Vazirianzadeh et al., 2014).

In general, many reliable laboratory methods are used to diagnose leishmaniasis, such as direct microscopy, culture, histopathology, immunologic and molecular techniques (de Vries et al., 2015; World Health Organization, 2020; World Health Organization, 2021). Currently, a reliable worldwide gold-standard single diagnostic test is not available for CL. The sensitivity of the diagnostic assays depends on the parasite load in the lesion (Aronson and Joya, 2019). Diagnosis of CL is difficult because it involves varied symptoms caused by different species. The management of CL differs from region to region and is primarily based on local experience-based evidence. *Leishmania* species vary in their sensitivity to available therapeutic options, and the choice of treatment method is critical for the clinical outcome (Dowlati, 1996; Murray et al., 2005). Optimized and validated techniques play an important role in diagnosis and accurate determination of infectious diseases (Dowlati, 1996; Murray et al., 2005). Currently, molecular epidemiology studies are known to assist in early diagnosis, treatment management, control and prevention of disease using advanced techniques (Parvizi et al., 2008; Fotouhi-Ardakani et al., 2016). PCR assay is known as a highly sensitive and specific test compared to other common methods for diagnosis and identification of *Leishmania* species and subspecies (Shahbazi et al., 2008; Bates et al., 2015; Eybpoosh et al., 2017). Various PCR methods have been described targeting multicopy and different parasite genes (Medina-Acosta et al., 1993; Nasereddin et al., 2009; Belinchón-Lorenzo et al., 2013). In PCR screening of smears from suspected cases (CL patients), up to 98.8% sensitivity have been reported while 79.3 and 86.2% sensitivities have been reported in microscopy and parasite culture methods, respectively (Shahbazi et al., 2008). Nested PCR (Ln-PCR) strategy has also been reported to increase sensitivity and specificity in *Leishmania* detection (Sreenivas et al., 2004; Maraghi et al., 2007; Parvizi et al., 2008; Azizi et al., 2010). The combination of PCR and restriction-fragment-length-polymorphism analysis (PCR-RFLP), especially using the ITS-1 region with the HaeIII Restriction Enzyme, is used for the detection and to distinguish *Leishmania* species (Dweik et al., 2007). Recently, quantitative real-time PCR (qPCR) assay has been developed as an acceptable and preferred technique for measuring parasite or microbial burden in clinical cases (Weirather et al., 2011; Hossain et al., 2017; Antonia et al., 2018; Xiu et al., 2020).

Among several genes that have been recognized for the discrimination of *Leishmania* species, two AAP3 and COII genes are useful for identifying many parasites across different geographical origins (Fotouhi-Ardakani et al., 2016; Aoki et al., 2017; Müller et al., 2018).

In this research, two new TaqMan probe-based qPCR assays were developed and validated by targeting COII and AAP3 gene regions for specific detection of *Leishmania* parasite and its common species (*L. major* and *L. tropica*) for the first time in Iran.

**Figure 1:**
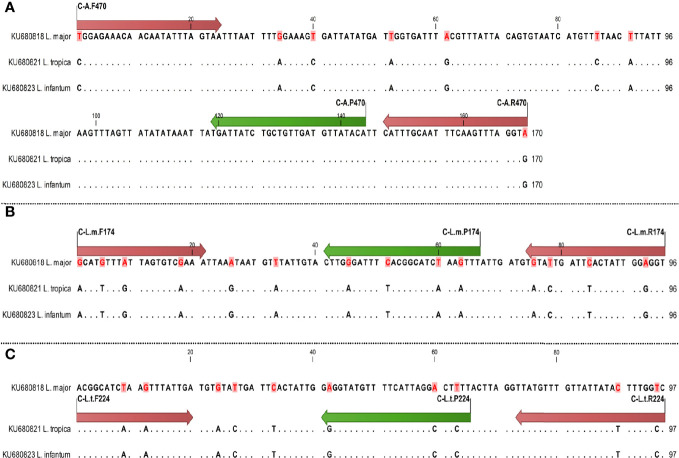
Schematic illustration of primers and probes (nucleotide site variation) in COII gene for diagnostics of *Leishmania* species using qPCR, arrows of start and end sites showing primer sites and internal arrows showing probe sites; difference of nucleotides is specified by dot. The primer and probe sites are shown by red and green arrows, respectively. Sequences are specified by dot except different nucleotides. **(A)** Specific primers and probes of all species; **(B)** specific primers and probes of *L. major*; **(C)** specific primers and probes of *L. tropica*.

**Figure 2:**
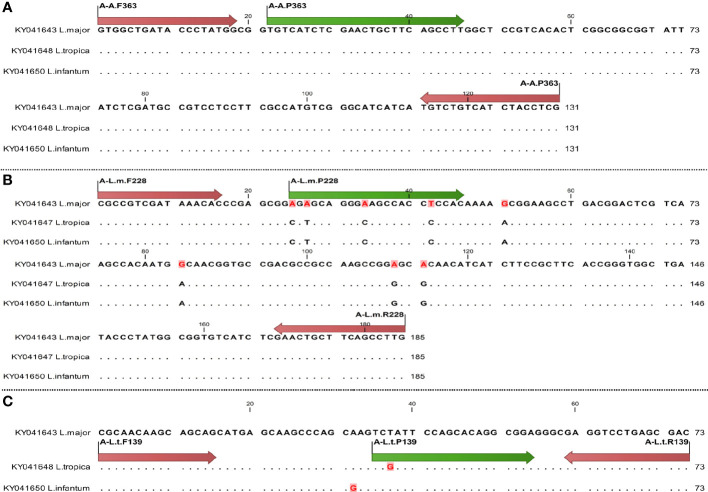
Schematic illustration of primer and probe (nucleotide site variation) in AAP3 gene for diagnostics of *Leishmania* species using qPCR, arrows of start and end sites showing primer sites and internal arrows showing probe site; difference of nucleotides is specified by dot. The primer and probe sites are shown by red and green arrows, respectively. Sequences are specified by dot except different nucleotides. **(A)** Specific primers and probes of all species; **(B)** specific primers and probes of *L. major*; **(C)** specific primers and probes of *L. tropica*.

In general, the purpose of this research was for firm identification and discrimination of *Leishmania* species as causative agents of leishmaniosis. To achieve this purpose, regarding high prevalence of the *Leishmania* parasite and availability of the specimens with specific identity, the specific objectives of this research were as follows: (1) Analyzing of two mitochondrial and nucleic genes using *in silico* software considering different functions, copy numbers, and practical natures (**Figures 1**, **2** and Fotouhi-Ardakani et al, 2016; Ghafari et al., 2020). (2) Designing of suitable primers and probes considering condition of conserved regions or with different genotyping in the implementation of the hard thermodynamic laws. (3) Predicting of primers and probes with medium and high risk considering optimization, validation, thermodynamic conditions for managing qPCR reactions.

## Materials and Methods

### Preparation of the Specimens

In total, 123 CL specimens were obtained including 98 clinical specimens, 12 sand flies gut and saliva in ethanol 70%, 9 specimens of captured rodents, and 4 reference strains of *Leishmania* parasites in NNN media. Ninety-eight clinical specimens (skin scraping/biopsy) were distinguished from microscopic slides including 7 in NNN media, 15 fresh specimens (non-cultured) in PBS for DNA extraction, and 76 specimens from last year’s archive.

Human samples consisted of 76 samples from Qom, 8 samples from Yazd, 3 samples from Kerman, 11 samples from Pasteur Institute of Iran with four reference strains. The reference strains included two World Health Organization (WHO) standard strains of *L. major* (MHOM/SU/73/5ASKH) and *L. tropica* (MHOM/SU/74/K27), and two other *L. major* reference strains (MRHO/IR/75/ER and MHOM/IL/80/friedlin) were obtained from the Department of Immunology, Pasteur Institute of Iran. Sand fly samples included seven samples from Yazd province and five samples from Khuzestan province, and rodent samples were collected from Khuzestan.

The collected samples were prepared by slit skin smears from the edge of the ulcer (Schönian et al., 2003; de Vries et al., 2015; World Health Organization, 2021). Microscopic assessments were carried out to determine the presence of amastigotes in all samples. Eleven samples (seven human samples and four reference samples) were cultured in NNN (Novy–McNeal–Nicolle) medium and were applied to promastigotes extraction. The cultures were set up at about 10 million promastigotes per ml in a Neubauer chamber.

### Analyzing of Gene Regions and Designing of Primers and Probes

AAP3 and COII genes were targeted in this research. To analyze the candidate gene regions, determine the genetic polymorphisms, and design specific primers and probes, *in silico* analyses of different regions of AAP3 and COII genes were performed and thermodynamic conditions were considered. Therefore, different bioinformatics tools were used, including CLC Genomics Workbench 12 (CLC, Bio-QIAGEN, Aarhus, Denmark) as well as Allele ID and Gene Runner 6.5.52 software (Kumar et al., 2016). Several reference sequences were derived from GenBank and loaded into the CLC Genomics Workbench 12 software. The best regions were selected for TaqMan-qPCR assay. Predictions of OligoAnalyser 3.1 software were considered to optimize the melting temperature (T_m_).

To achieve the goal of internal control construction, an innovative gene structure was designed and synthesized (**Supplementary Figure 1**).

### DNA Extraction, Amplification, and Sequencing

After precipitation of purified promastigotes (10×10^6^ cells/ml, late-log phase), all DNA samples were extracted using CinnaPure DNA Kit (CinnaClon, Iran) following the manufacturer’s instructions. PCR was applied: genomic DNA 10 ng in a final volume of 25 μl containing PCR buffer 1× [750 mM of Tris-HCl, pH: 8.8 at 25°C, 200 mM of (NH_4_)_2_SO_4_ and 0.1% Tween-20], 2.5 mM of MgCl_2_, 0.5 mM of deoxyribonucleotide triphosphate (dNTP), 1 U of Taq polymerase (CinnaClon, Iran), and 0.4 pmol of each primer (Tellevik et al., 2014; Fotouhi-Ardakani et al., 2016). The PCR reaction was subjected to 35 cycles of denaturation at 94°C for 30 s, annealing for each gene (COII: at 45°C for 45 s; AAP3: at 58°C for 45 s), and extension at 72°C for 1 min and final extension at 72°C for 5 min. After purification, the PCR products were sequenced using an ABI PRISM™ 310 automated sequencer (Applied Biosystems, USA), using specific forward and reverse primers (Ghafari et al., 2020).

### Cloning and Making Standard Criteria

In different species of parasite (*L. major* and *L. tropica*), selection of diagnostic domains was done to construct the standard targets for both genes (COII and AAP3). PCR products were cloned directly into the pTG19-T vector by the TA cloning method (PCR TA Cloning kit, CinnaClon, Iran). Serial dilution of purified plasmid was used as standard criterion for determining the copy number, efficiency, and regression ratio of optimized reactions.

### Developing of the Multiplex qPCR Quantification

TaqMan-qPCR reaction was developed to detect *L. major* and *L. tropica* species. Briefly, a 20 μl reaction mix was prepared containing 2 µl template [10 ng/reaction (rxn)], 10 µl RealQ Plus 2X Master Mix for Probe without ROX™ (AMPIQON, Denmark), 0.4 pmol of each primer and 0.3 pmol of each probe, and 5.8 µl of PCR grade water. Amplification was performed by RotorGene 6000 qPCR (Corbett Life Science, Australia) following these conditions: 15 min at 95°C, followed 45 cycles of 20 s at 94°C, annealing for each gene (COII: at 55°C for 60 s, AAP3: at 60°C for 60 s).

To quantify the parasite load of each sample, each run included one standard curve with DNA concentrations corresponding 1 to 1,000,000 parasites per reaction. Each run also included one reaction with molecular grade water as a negative control. Each DNA sample was evaluated in triplicate. Samples with cycle threshold (CT) >40 were considered negative (Ghafari et al., 2020).

### Analytical Sensitivity and Specificity, Linearity and Reproducibility of TaqMan-qPCR

In accordance with the parasitic load of the genesig^®^ *Leishmania* Advanced Kit standards, a serial dilution 1:10 of purified plasmid (2–2×10^7^ copies/rxn) was prepared under the same conditions for both genes. All dilutions were assayed in triplicate. To determine the limit of detection (LOD) of the parasite, six replicates of twofold DNA serial dilution, with concentration of 200 to three parasites per reaction, were evaluated. The Primer-Blast tool was used to assess the specificity of designed primers and probes in both genes.

Linear range dilutions (20–2×10^6^ copies/rxn) were prepared to evaluate and determine precision or reproducibility in both genes. Inter-assay and intra-assay validations were performed for 2 days by two users using three replicates on each standard with two RotorGene and Biosystems StepOnePlus™ (CA, USA) machines. Precision data were calculated based on the devices’ threshold cycle (CT) values. The coefficient of variation was used to assess the variability of quantitative assays [CV represented the ratio of mean to standard deviation (SD)].

### Clinical Sensitivity, Specificity, and Statistical Analysis

The clinical sensitivity and specificity of this assay were calculated to evaluate the performance and quality of the test in the detection and identification of *Leishmania* parasite. The receiver operating characteristic (ROC) test was employed to compare the CT values of TaqMan- qPCR assay using COII and AAP3 genes and the genesig^®^ *Leishmania* Advanced kit, in order to improve the sensitivity and specificity.

## Results

### Microscopic Examination

DNA of all 123 samples were extracted (112 fixed slides first identified with microscopic assessment then applied to DNA extraction) as well as the promastigotes extracted from 11 cultured samples in NNN medium (seven human and four reference samples). Seventy-seven out of 123 were *Leishmania*-positive and 46 were negative.

### AAP3 and COII Genes Multiplex Amplification and Molecular Analysis

Three regions of each gene (AAP3 and COII) were targeted to separate and diagnose the *Leishmania* species. The COII gene was more sensitive than AAP3, but AAP3 was better in terms of protection and GC percentage for discrimination and separation of *Leishmania* species (**Table 1**). The fragments of AAP3 (476 bp) and COII (611 bp) were amplified by PCR. The PCR products were sequenced and submitted to GenBank. The GenBank accession numbers of AAP3 and COII genes were KY041643-KY041649 (available at https://www.ncbi.nlm.nih.gov/nuccore/KY041643toKY041649) and KU680818-KU680821 (Fotouhi-Ardakani et al., 2016; Ghafari et al., 2020), respectively. Among 123 DNA samples, 77 were *Leishmania*-positive samples identified using both microscopic examinations and PCR. Forty-six samples were negative in terms of the presence of *Leishmania* and were diagnosed as bacterial and fungal infections or normal eczematic dermatitis. In other words, leishmaniasis was not the cause of ulcers in these patients. Out of 77 *Leishmania*-positive samples, 58 were identified as *L. major* and 19 samples as *L. tropica*. All species of *Leishmania* identified in rodents and sand flies were *L. major*. But in the human clinical samples, both *L. tropica* and *L. major* were identified (Ghafari et al., 2020).

**Table 1:** Comparison and validation of repeatability results of two COII and AAP3 genes.

Genes	Dilutions (20–2×10^6^)	RotorGene instrument Intra-assay CT values			StepOne Plus instrument Inter-assay CT values		
		Mean CT	SD	CV	Mean CT	SD	CV
**COII**	20	30.13	0.576	1.913	31.45	0.638	2.029
	200	25.02	0.125	0.502	28.15	0.264	0.939
	2,000	21.84	0.24	1.099	25.55	0.4	1.565
	20,000	18.81	0.223	1.186	22.15	0.251	1.136
	200,000	14.88	0.079	0.535	21.55	0.253	1.175
	2,000,000	12.35	0.077	0.624	17.43	0.261	1.501
	Mean	20.50	0.22	0.97	24.38	0.34	1.39
**AAP3**	20	30.54	0.576	1.888	32.45	0.638	1.967
	200	26.02	0.125	0.483	29.15	0.412	1.102
	2,000	23.75	0.240	1.010	25.55	0.305	1.195
	20,000	20.45	0.223	1.091	23.15	0.432	1.422
	200,000	17.38	0.079	0.458	20.81	0.477	1.302
	2,000,000	14.98	0.088	0.592	15.43	0.542	2.066
	Mean	22.186	0.22	0.92	24.42	0.36	1.967
**Validation**		**Slop**	**R^2^**	**Efficiency**	**Slop**	**R^2^**	**Efficiency**
**COII**		−3.49	0.99	0.93	−2.66	0.98	1.17
**AAP3**		−3.0577	0.99	1.12	−3.21	0.99	1.01

The precision of results was compared in different dilutions using C-A.P470 and A-A.P363 probes in L. major species employing two real-time PCR instruments.

CT, Cycle of Threshold; SD, Standard Deviation; CV, Coefficient of Variation.

This gene structure contains 76 nucleotides; 23 nucleotides of the end of 3 placed as a specific reverse primer for COII gene. The remaining 56 nucleotides were designed as a stem-loop which denaturant at 60°C and were amplified with a primer and probe on the HEX channel (**Supplementary Figure 1**).

### Validation of New Designed Primers and Probes

Six sets of primers and probes were designed to optimize and validate TaqMan-qPCR assay for both AAP3 and COII genes (**Table 2**). Two sets of common primers and probes (C-A.470 and A-A.363) were designed for all *Leishmania* species in both COII and AAP3 genes. The *Leishmania* specific primers and probes were selected and designed from conserved regions in both genes, which well identified both *L. major* and *L. tropica* species. The competitive binding of non-specific primers and probes led to the differentiation of different *Leishmania* species. The primer and probe sets of C-L.m.174 and C-L.t.224 for COII gene and two sets of A-L.m.228 and A-L.t.139 for AAP3 gene were specific for *L. major* and *L. tropica*, respectively.

**Table 2:** Designed primers and probes in TaqMan real-time PCR method for *Leishmania* species in this study.

Fragments length bp	T_m_* °C	Primers and probes sequences (5’ -> 3’)	Primers and probes	Species	Genes
170	61.2	FAM-TGTATAACATCAACAGCAGATAATCA-BHQ1	C-A.P470	All species	**COII**
	55.3	YGGAGAAACAACAATATTTAG	C-A.F470		
	55	YACCTAAACTTGAAATTGC	C-A.R470		
96	65.5	FAM-AAACTTAGATGCCGTGAAATCCCAAG-BHQ1	C-L.m.P174	*L. major*	
	57.2	GCATGTTTATTAGTGTCGAAA	C-L.m.F174		
	58.5	ACCTCCAATAATGAATCAATACA	C-.L.m.R174		
97	64.7	Texas Red-AAGAGGCCTAATGAAAACATACCCC-BHQ2	C-L.t.P224	*L. tropica*	
	56.8	ACGGCATCAAAATTTATTGA	C-L.t.F224		
	57.6	GGCCAAAATATAATAACAAACATAA	C-L.t.R224		
131	67.8	FAM-TGTCATCTCGAACTGCTTCAGCCTT-BHQ1	A-A.P363	All species	**AAP3**
	58.5	GTGGCTGATACCCTATGG	A-A.F363		
	57.4	CGAGGTAGATGACAGACA	A-A.R363		
185	69.3	FAM-AGAGCAGGGAAGCCACCTCCA-BHQ1	A-L.m.P228	*L. major*	
	57.2	CGCCGTCGATAAACAC	A-L.m.F228		
	57.7	CAAGGCTGAAGCAGTTC	A-L.m.R228		
73	67.9	Texas Red-TCGATTCCAGCACAGGCGGA-BHQ2	A-L.t.P139	*L. tropica*	
	58	CGCAACAAGCAGCAG	A-L.t.F139		
	57.3	GTCGCTCAGGACCTC	A-L.t.R139		

The designation was done with in silico analyses of different regions of COII and AAP3 genes and after consideration of thermodynamic conditions.

^*^Melting Temperature Settings in OligoAnalyser 3.1, qPCR defaults.

The specific sets of *L. major* with *L. tropica* species differed between one to four nucleotides (**Figures 1**, **2**). The melting temperature differences (T_m_) were evaluated based on the amplified fragments length, primers size, and their nucleotide differences (**Table 2**).

### Analyzing Sensitivity and Specificity of TaqMan-qPCR

**Figure 3:**
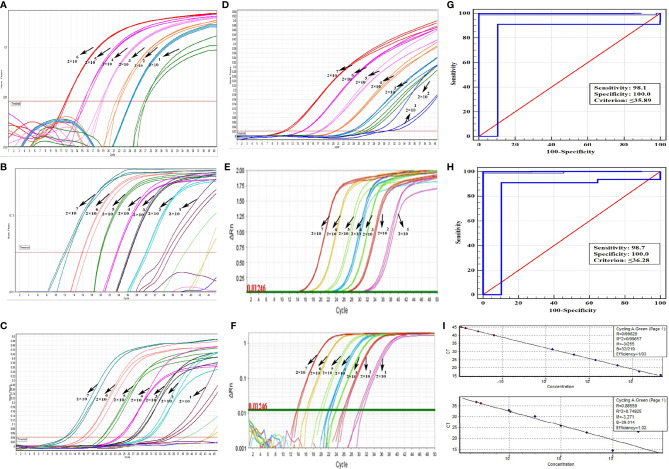
Performance specifications of *Leishmania* detection with qPCR validation. Amplification plots derived from serial dilutions of cultured parasites, ranging from 20 to 2 × 10^7^ copies/rxn plasmids by qPCR. FAM fluorescent reporter dye of COII gene, compared to Cyt b (FAM fluorophore) in the reference kit was displayed. **(A)** Reportable range of *Leishmania* (20–2 × 10^6^). **(B, C)** Logarithmic and linearity amplification plots of detection limit of *Leishmania* (20–2 × 10^7^) using COII, respectively. **(D)** Linearity amplification plot of detection limit of *Leishmania* using Cyt b in reference kit. **(E, F)** Linearity and logarithmic amplification plot of quantification limit of reproducibility using StepOnePlus™ machine, respectively. Amplification curves are shown for each sample, with each parasite concentration depicted by different color. **(G)** Clinical sensitivity and specificity of *Leishmania* diagnostic by ROC analysis (sensitivity = 98.1, diagnostic CT = 35.89) using COII. **(H)** Clinical sensitivity and specificity of *Leishmania* with genesig^®^ *Leishmania* Advanced diagnostic kit (sensitivity = 98.7, diagnostic CT = 36.28) using Cyt b **(I)** Standard quantification curves, the mean CT values are plotted from triplicates tested against prepared serial dilutions. Each point represents CT of an individual sample, with plot of CT values and parasite equivalent fitting a linear function (R^2^ = 0.996 for qPCR and, R^2^ = 0.749 for genesig^®^ *Leishmania* Advanced kit) (See **Table 2**).

The specificity of the designed primers and probes was confirmed by BLAST analyses. In both genes, the lower limit of quantification (LLOQ) and the upper limit of quantification (ULOQ) were 20 and 2×10^6^ copies/rxn plasmids, respectively (**Figure 3A**). Although in our experiments less than this amount was separable, the efficiency of the reaction became very low. The *Leishmania* specific primers and probes of C-A.470 and A-A.363 were able to detect six and three copies of the parasite in the COII and AAP3 genes, respectively. Specific primers and probes of the *L. major* and *L. tropica* species were able to detect three copies of the parasite, but for non-specific species, they were not able to amplify in dilutions lower than three copies (**Figures 3B–D**).

Reproducibility and within-run imprecision were assessed as intra-assay variation of CT values for six series dilutions of both genes using C-A.P470 and A-A.P363 probes for *L. major* species by employing RotorGene machine. In intra-assay validation, the mean coefficient of variations (CV) were 0.97 and 0.92 for the COII and AAP3 genes, respectively. Inter-assay variation (assessment of reproducibility run-to-run and day-to-day imprecision) was investigated using the same probes and StepOnePlus™ machine. In both COII and AAP3 genes, the mean CV of inter-assay of six different concentrations was found 1.39 and 1.967 (**Table 1** and **Figures 3A, E, F**).

The analysis of ROC curves showed that only one of 77 positive samples were negative using all three *Leishmania* TaqMan-qPCR assays. The sensitivities of the reference kit and our methods were 98.7 and 98.1%. The diagnostic CT of the reference kit and this assay was 36.28 and 35.89, respectively. All 46 negative samples were confirmed by PCR. Specificity was observed as 100% in qPCR assays (**Figures 3G, H**).

The primers and designed probes performed well in terms of efficiency and regression coefficient. In standard generated curves, the linear (y = −3.25x + 32.21 and y = −3.27x +39.014) showed a 7 log range with correlation coefficients (R^2^) of 0.996 and 0.749 for our assay and genesig^®^ kit, respectively, by serial dilutions of parasite DNA (**Figure 3I**).

### Comparing Performance Specifications and Validation of TaqMan-qPCR Assays

Calibration curves of absolute plasmid DNA standards (2–2 × 10^6^) were developed to evaluate the performance specifications of qPCR assays using thelinear regression analysis of associated CT measurements. TaqMan-qPCR-based calibration curves were compared to each other using the regression coefficients arising from the gradient or slope between two regression lines.

Then, the amplification efficiency of the qPCR assays was determined by the regression coefficient of each reaction.

For better coordination of the methods with the genesig^®^ kit, the Cyt b gene standards were used as the diagnostic standard with the primers and probes of the genesig^®^ kit as well as new designed primers and probes.The amount of standard plasmids of Cyt b, COII, and AAP3 genes was calculated and adjusted according to the quantitation formula of the genesig^®^ kit. Regression coefficients obtained from six Cyt b standards of the genesig^®^ Kit and the Cyt b standard plasmids with linear regression equations of y = −3.2193x + 35.017 and y = −3.2825x + 34.847 showed efficiency of 1.04 and 1.01 with R^2^ of 0.997 and 0.983, respectively.

In calibration curves produced by two linear regressions based on the standards of COII and AAP3 genes using the specific primers and probes of C-A.P470 (COII gene) and A-A.P363 (AAP3 gene), efficiency showed 0.904 with the linear equation of y = −3.5754x + 32.857 and R² = 0.97 for the COII gene, while the efficiency was obtained 1.01 with linear equation of y = −3.2825x + 34.847 and R² = 0.98 for the AAP3 gene. The precision of the qPCR methods was established using repeatability studies by comparing the imprecision association between two reactions in two different qPCR machines.

For the AAP3 gene, the mean CV was 0.92 and 1.967 in the RotorGene and the StepOne Plus machines. The comparison of their averages did not show any difference between two machines (P = 0.06). In evaluating the precision of qPCR assays with the RotorGene and StepOne Plus machines using the AAP3 standards, regression coefficient for the RotorGene machine showed efficiency of 1.12 with linear equations of y = −3.0577x + 33.809 and R² = 0.98, while the efficiency was obtained 1.01 with linear equation of y = −3.2149x + 36.643 for the StepOne Plus machine.

For the COII gene, the mean CV was 0.97 and 1.39 in the RotorGene and StepOne Plus, respectively. The comparison of CV mean for this gene showed no significant difference between RotorGene and StepOne Plus (P = 0.16) (**Table 1**). Regression coefficients of RotorGene and StepOne Plus machines showed efficiency of 1.17 in the StepOne Plus machine with linear equation of y = −2.6657x + 34.512 and R^2^ of 0.98, while the efficiency was obtained 0.93 with linear equation of y = −3.4957x + 33.792 and R^2^ of 0.99 for the RotorGene machine.

### Correlation of Patients’ Quantification Using Three qPCR Assays

**Figure 4:**
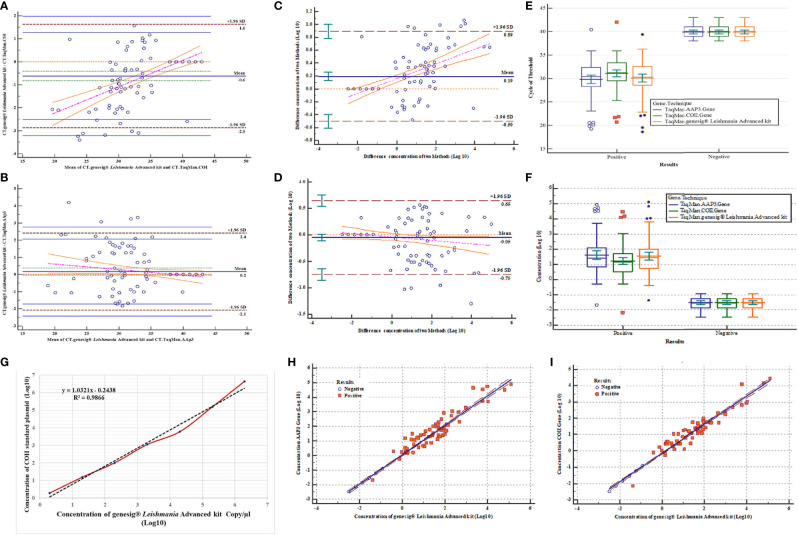
Assessment of validation results and correlation of three qPCR assays. **(A)** Bland-Altman test for comparison of mean CT difference of 123 samples (77 positives and 46 negatives) for COII gene with genesig^®^ *Leishmania* Advanced Kit. **(B)** Bland-Altman test for comparing of mean CT difference of 123 samples for AAP3 gene with genesig^®^ *Leishmania* Advanced Kit. **(C)** Bland-Altman test for comparing of concentration differences (log10) of 123 samples for COII with genesig^®^ *Leishmania* Advanced Kit. **(D)** Bland-Altman test for comparing of concentration differences (log10) of 123 samples for AAP3 with genesig^®^ *Leishmania* Advanced Kit. **(E)** Comparison of mean CT difference plasmids of AAP3 and COII and genesig^®^ *Leishmania* Advanced kit based on negative and positive samples. **(F)** Comparison of concentration differences (log10) plasmids of AAP3, COII, and genesig^®^ *Leishmania* Advanced kit based on negative and positive samples. **(G)** Comparison of linear regression analysis of COII plasmid standards and genesig^®^ kit by linear equation (y = −3.2193x + 35.017) obtained from genesig^®^ *Leishmania* Advanced kit. **(H)** Dispersion diagram with regression line to compare results of parasitic load concentration of genesig^®^ *Leishmania* Advanced Kit assay with AAP3 for 123 samples. **(I)** Dispersion diagram with regression line to compare results of parasitic load concentration of genesig^®^ *Leishmania* Advanced Kit assay with COII for 123 samples.

Comparison between the results of three methods showed that mean CT difference of only one sample was at the lower limit of two standard deviations by the Bland-Altman test for the COII gene. In AAP3 gene, four samples were at the upper limit of two standard deviations. Also, logarithmic comparisons of concentration difference for the COII gene showed that there were four samples at the lower limit, while for the AAP3 gene, only one sample was at the upper limit of two standard deviations (**Figures 4A–D**).

Parasitic load variance of three assays was evaluated using two-way ANOVA test based on the detection limit for positive and negative samples with their Clustered Multiple Comparison graph drawn. Comparison of means and their differences showed no significant difference in CT mean and parasitic load between three assays by ANOVA test (P > 0.05) (**Figures 4E, F**). The evaluation results of the calculated parasitic load concentration of COII gene (estimated with the genesig^®^ kit) showed the linear equation of y = −3.2193x + 35.017, confirming the high correlation between assays (R^2^ = 0.986) (**Figure 4G**). To investigate the linearity of the assays, association analyses were employed using Scatter diagrams and Regression tests. All samples showed good and statistically significant associations, with the samples widely spread in the reportable range of the tests for the two new designed assays. For AAP3 and COII genes, with linear equations of y = 1.013x − 0.612 and y = 0.87x − 0.145, the observed regression coefficients were 0.98 and 0.96 with slopes of 1.013 and 0.87, respectively (**Figures 4H, I**).

## Discussion

In the last 10 years, qPCR techniques have been developed with more time-efficient and sensitive advantages for diagnosis and accurate identification of *Leishmania* parasites (Belinchón-Lorenzo et al., 2013; Tellevik et al., 2014; Hossain et al., 2017; Antonia et al., 2018).

In the current research, optimization and validation of TaqMan-qPCR assays were performed to develop a diagnostic kit for qualitative and quantitative detection of *Leishmania* regional species.

This technique is a multiplex qPCR since it employs different sets of primers and probes to detect simultaneously different targets, and it is possible to discriminate between the targets only employing qPCR.

The advantages of applying the TaqMan real-time PCR technique compared to more cost-effective molecular techniques (such as PCR-RFLP and nested PCR) include greater sensitivity, time-efficiency, and faster detection, and this applies to a range of diseases including cancers (Oh et al., 2009). Selection of a suitable target gene region, the copy number of the target gene, and the specimen type have a great impact on the quality of qPCR validation. In addition, the sensitivity of the CL diagnostic assays depends on the parasite load in the ulcer biopsies and/or diagnostic slides (Ranasinghe et al., 2008; Weirather et al., 2011; Hossain et al., 2017; Antonia et al., 2018). Usually, more sensitive gene regions such as minicircle kDNA and ITS2 genes were used to detect *Leishmania* parasites in separate reactions (Mary et al., 2004; Cavalcanti et al., 2009; Fekri Soofi Abadi et al., 2016).

In homemade developed tests in the medical laboratories, three objects have to be considered in the validation of the tests, such as *Leishmania*, *Corona*, and *Influenza*: (1) The wrong percentages of the false negative with LOD research have to be determined concerning the principles of analytical sensitivity. The false positive has to be determined with careful research on cross-reaction probability according to analytical specificity principles. This research reached to this important conclusion in *Leishmania* parasite detection with determining the number of cultured parasite as well as normalizing the reaction conditions of two cloned genes in the plasmid, for the first time.

(2) The accuracy or repeatability has to be assessed and specified, in different examination conditions and different instruments. Meanwhile, this research determined comparison of the results in two instruments and two genes’ accuracy in normalized conditions with Cyt b in reference kit.

(3) Applying the same method in the determination of the results’ correctness, for absolute quantification, is one of important achievements in the manufacturing of molecular kits, so that the fragment Cyt b was cloned and the primer and probes of the genesig^®^ kit confirmed. Cyt b gene was coincided to targeted genes. In this study, for similarization of quantity detection in the determination of the copy numbers of the targeted gene with this kit, the large fragment of the gene was cloned. After determination of gene copy numbers, it was applied for the comparison to the targeted genes.

Based on bioinformatics studies of different gene regions, we investigated conserved areas with genetic diversity suitable for the design of primers and specific probes for the detection of different species of *Leishmania*. For this purpose, two COII and AAP3 genes were targeted. The diagnostic ability of the COII gene (10–20 copies in each mitochondrion) and the AAP3 gene (two copies in the parasite genome) differ in *Leishmania* (Tellevik et al., 2014; Fotouhi-Ardakani et al., 2016). In TaqMan-qPCR assays, primers play an important role in the hydrolysis probe. Using and designing several primers enables the detection of different *Leishmania* species. The specificity of the probe, especially at the 5’ end, affects the thermodynamic importance of the probe and enhances the reaction efficiency (Nagy et al., 2017; Taylor et al., 2019).

For COII and AAP3 genes, six new sets of primers and probes were designed to isolate and identify *Leishmania* parasite using TaqMan-qPCR assay. Two sets of newly designed primer and probe (C-A.470 and A-A.363) were used for all species. Also, species-specific primers and probes were designed for both genes which were non-specific for other species (Didelot et al., 2012). Different species of *Leishmania* were discriminated using competitive combinations of non-specific primers and probes (Weirather et al., 2011). Two sets of primer and probe named C-L.m.174 and C-L.t.224 for the COII gene and two sets of A-L.m.228 and A-L.t.139 for the AAP*3* gene identified *L. major* and *L. tropica*, respectively. The two *L. major* and *L. tropica* species are the causative agents of almost all CL in Iran. Amplification of innovative internal control was designed to prevent false negatives.

Microscopic assessment is the cheapest and easiest method to determine the presence of *Leishmania* amastigotes in the clinical samples but is time-consuming and has less sensitivity compared with qPCR (Dowlati, 1996; Desjeux, 2004; Hossain et al., 2017; World Health Organization, 2021). From 123 prepared specimens in this study, 77 positive cases were identified using both microscopic examination and PCR.

Evaluation of the parameters of accuracy, precision, reportable range, reference range, analytical specificity-sensitivity, and other required indicators is essential for validation of developed tests. To identify *Leishmania* (all species) by targeting Cyt b kinetoplast gene, qPCR results were compared with Primerdesign™ Ltd genesig^®^ Advanced Kit findings. Preparation of diagnostic standards with a specified copy number is required to determine the parasitic load and primer specificity (Schaad and Frederick, 2002; Piña-Vázquez et al., 2008; Weirather et al., 2011).

For both genes, six diagnostic standards were prepared according to the number of plasmid copies of the reference kit. Serial dilutions of (20–2×10^6^ copies/rxn) of purified plasmids showed that low limit of quantification was 20 copies within the linear range of optimized assays. Although less than 20 copies were separable, the efficiency of the reaction was very low. Another study showed this linear range is from 10 to 10^7^ copies (Fekri Soofi Abadi et al., 2016).

This developed assay offered high specificity. The analytical and diagnostic specificity of both assays were evaluated. The analytical specificity was well confirmed with designed primers and probes in multiple hosts at NCBI (non-specific product not shown). The diagnostic specificity of both TaqMan-qPCR assays was 100% as all 46 negative samples were negative. The assays in this study have been more specific than others (Cavalcanti et al., 2009; Fekri Soofi Abadi et al., 2016). In addition, the analytical and diagnostic sensitivity of both assays was evaluated. Both assays were highly sensitive with a reliable limit of detection (three copies of the parasite). *Leishmania*-specific primers and probes were able to detect six and three copies of the parasite in COII and AAP3 genes, respectively. The diagnostic CT of the reference kit and these assays (36.28 and 35.89) showed that the COII gene was a useful target despite its high copy number.

The analysis of ROC curves showed that only one out of 77 positive samples could not be detected by qPCR assays; meanwhile, the diagnostic sensitivity of the reference kit and our assays was 98.7 and 98.1%. Thus, these assays were less sensitive than those of other studies (Hossain et al., 2017). The most important reason could be old slide samples, from our last year’s archive low parasite loads, and/or low efficiency of DNA extraction (Cavalcanti et al., 2009; Fekri Soofi Abadi et al., 2016). Reproducibility is one of the important parameters for estimating the imprecision rate or random error of the test in the study of a laboratory method. The precision of qPCR method was established by repeatability studies for both AAP3 and COII genes. The imprecision association between two reactions in two different qPCR machines was compared.

The RotorGene machine with lower mean CVs showed higher precision than the StepOnePlus™ machine. Thus, the efficiency of reaction was higher and better in the RotorGene (1.12 *vs.* 1.01 and 1.17 *vs.* 0.93) for AAP3 gene and COII gene, respectively. The mean comparison of CVs showed no significant difference either between the two machines (P=0.06 and P = 0.16 for AAP3 and COII, respectively).

On the other hand, the competitive performance of species-specific primers and probes depends on the probe location and number of nucleotide differences leading to different reactions. Efficiency and R^2^ coefficient of reactions with the optimized standards are the best criteria for different species differentiation (Ramakers et al., 2003; Real-Time PCR handbook, 2014). Reaction efficiency of AAP3 indicated no difference in the precision of assays, while the efficiency of AAP3 reaction was higher due to better performance of the probe.

Proximity and agreement between data obtained from study and reference methods are essential to verify the accuracy of the reaction. The interpretation of data correlation is useful for both statistical and graphical annotation. The most common method for suitable evaluation and analysis of agreement between two quantitative methods is mean difference diagram described by Giavarina (Giavarina, 2015). Comparison between means and their differences revealed no significant difference in the CT mean and parasitic load among three assays using ANOVA test (P>0.05). The dispersion diagrams showed no significant difference either in the correlation between two new designed and reference assays. They were widely distributed in the reportable range of the test.

Finally, two new TaqMan probes based on qPCR assay were developed and validated for the specific detection of *Leishmania* parasite and its common species plus strains (*L. major* and *L. tropica*) in Iran. Validation experiments were performed to ensure the accuracy and experimental transparency of the development of the new assays. The validation results showed that the assays are not only highly specific and sensitive but also reproducible and highly accurate. Two new assays can serve as an additional tool for early detection and confirmation of *Leishmania* infection.

## Data Availability Statement

The datasets presented in this study can be found in online repositories. The names of the repository/repositories and accession number(s) can be found in the article/**Supplementary Material**.

## Ethics Statement

The studies involving human participants were reviewed and approved by clinical sample collection followed the Declaration of Helsinki, Ethical Principles for Medical Research Involving Human Subjects protocol, and Ethical Principles of Committee Guidelines of Pasteur Institute of Iran, No. IR.PII.REC.1398.050. The patients/participants provided their written informed consent to participate in this study. The animal study was reviewed and approved by clinical sample collection followed the Declaration of Helsinki, Ethical Principles for Medical Research Involving Human Subjects protocol, and Ethical Principles of Committee Guidelines of Pasteur Institute of Iran, No. IR.PII.REC.1398.050.

## Author Contributions

Conceptualization: RA, PP, SG, and PR. Data curation, investigation, methodology, software, and visualization: RA and SG. Roles/Writing—original draft and writing—review and editing: RA, PP, SG, and PR. Formal analysis and validation: RA. Funding acquisition and project administration, Resources and supervision: PP. All authors contributed to the article and approved the submitted version.

## Funding

This work was supported by the Pasteur Institute of Iran (grant number 735); a research fund granted by the National Institute for Medical Research Development (NIMAD) (Project No. 973166) awarded to PP; and Qom University of Medical Sciences (grant number IR.MUQ.REC.1397.063).

## Conflict of Interest

The authors declare that the research was conducted in the absence of any commercial or financial relationships that could be construed as a potential conflict of interest.

## Publisher’s Note

All claims expressed in this article are solely those of the authors and do not necessarily represent those of their affiliated organizations, or those of the publisher, the editors and the reviewers. Any product that may be evaluated in this article, or claim that may be made by its manufacturer, is not guaranteed or endorsed by the publisher.
