# Supplementary figures and images for: Developing, Modifying, and Validating a TaqMan Real-Time PCR Technique for Accurate Identification of Leishmania Parasites Causing Most Leishmaniasis in Iran
Source: Front Cell Infect Microbiol. 2021 Oct 12;11:731595. doi: 10.3389/fcimb.2021.731595 (PMC8546265; doi:10.3389/fcimb.2021.731595)

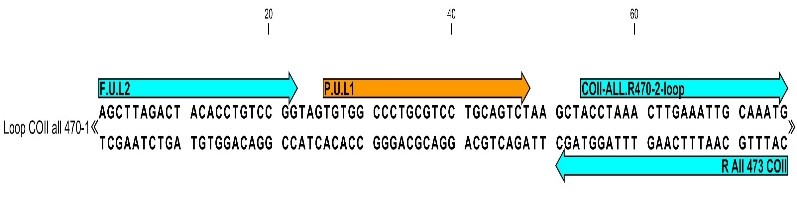

Supplement: Supplementary file 1 [file Image_1.jpeg]
