# Supplementary material for: Developing, Modifying, and Validating a TaqMan Real-Time PCR Technique for Accurate Identification of Leishmania Parasites Causing Most Leishmaniasis in Iran
Source: Front Cell Infect Microbiol. 2021 Oct 12;11:731595. doi: 10.3389/fcimb.2021.731595 (PMC8546265; doi:10.3389/fcimb.2021.731595)
Supplement: Supplementary file 2 [file Table_1.docx]

Supplementary Table 1. Validation of all *Leishmania* parasites (*L. major* and *L. tropica*) were sampled in different hosts and regions of Iran using Real-time PCR.

| **Origin**  **Species** | **IPI*** | **Qom**  **(slide)** | **Yazd**  **Abarkoh** | **Khuzestan** | **Kerman**  **Bam** | **Reference**  **strain** | **Total** |
| --- | --- | --- | --- | --- | --- | --- | --- |
| ***L. major*** | **0** | **28** | **13** | **14** | **0** | **3** | **58** |
| ***L. tropica*** | **0** | **13** | **2** | **0** | **3** | **1** | **19** |
| **Negative** | **11** | **35** | **0** | **0** | **0** | **0** | **46** |
| **Total**  **Host** | **11** | **76** | **15** | **14** | **3** | **4** | **123** |
| **Human** | **11** | **76** | **8** | **0** | **3** | **4** | **102** |
| **Sand-fly** | **0** | **0** | **7** | **5** | **0** | **0** | **12** |
| **Rodent** | **0** | **0** | **0** | **9** | **0** | **0** | **9** |
